# Cultural identity as a determinant of caregiving burden and quality of life in indigenous family caregivers of people with schizophrenia

**DOI:** 10.3389/fpsyg.2026.1750195

**Published:** 2026-03-12

**Authors:** Alejandra Caqueo-Urízar, Lizeth Carrillo-Alave, Felipe Ponce-Correa

**Affiliations:** 1 Instituto de Alta Investigación, Universidad de Tarapacá, Arica, Chile; 2 Escuela de Psicología y Filosofía, Universidad de Tarapacá, Arica, Chile

**Keywords:** caregiver burden, indigenous healing, Latin America, quality of life, schizophrenia

## Abstract

**Introduction:**

The study aimed to examine the relationship between ethnic background, caregiver burden, and quality of life among caregivers of individuals withSchizophrenia in an intercultural context.

**Methods:**

A cross-sectional descriptive-comparative design was used with one hundred seventy-five caregivers recruited from public mental health centers in Northern Chile, who completed standardized assessments of burden, quality of life, and sociodemographic characteristics. Statistical analyses compared Aymara and non-Aymara caregivers and evaluated the interaction between ethnicity and sociodemographic factors.

**Results:**

The findings showed no significant differences between ethnic groups in overall burden or global quality of life, although Aymara caregivers reported greater psychological strain in daily activities and non-Aymara caregivers reported better partner relationships. Interaction effects indicated that ethnicity combined with sex, partner status, religious affiliation, and patient age influenced certain dimensions of quality of life, particularly relationships with the psychiatric team and material strain.

**Discussion:**

These results suggest a convergence in caregiving experiences across ethnic groups, likely shaped by intercultural health policies, social integration, and cultural resilience. The study highlights the importance of culturally sensitive mental health services that incorporate Indigenous worldviews and strengthen support for family caregivers.

## Background

Schizophrenia, as one of the most severe and chronic mental disorders, has a profound impact on the lives of affected individuals and their families. The responsibility of caring for these patients falls largely on their relatives, which positions caregivers as central figures in daily support and supervision (Pan et al., 2024). This role involves performing medical, domestic, and emotional tasks, as well as managing behavioral disorganization, relapses, and experiences of social stigma. These demands often generate a set of tensions conceptualized as caregiver burden, understood as the collection of negative responses, whether objective or subjective, that arise from assuming long-term responsibility for another person’s well-being (Liu et al., 2020).

Objective burden refers to the concrete demands of caregiving, while subjective burden reflects the caregiver’s emotional experience, which may include stress, anxiety, depressive symptoms, social isolation, and feelings of helplessness (Gagiu et al., 2025). Several factors, such as the sex of both patient and caregiver, family relationship, age, educational level, economic resources, and duration of caregiving, influence the magnitude of this burden (Yu et al., 2018; Rahmani et al., 2022; Qiu et al., 2023). Illness severity, the presence of problematic behaviors, affiliate stigma, and low social support also increase caregiver vulnerability and negatively affect overall well-being and quality of life (Batista et al., 2023). In addition, a greater number of hours dedicated to caregiving, low educational attainment, and recent patient hospitalization appear to significantly deteriorate caregiver well-being (Al-Awad, 2024; Lima-Rodríguez et al., 2022).

Caregiver quality of life, defined as the individual’s perception of their position in life in relation to their expectations and cultural context (Munie et al., 2024), is generally lower than that of caregivers of individuals with other chronic conditions (Kojima et al., 2024). Several studies indicate that female caregivers, particularly mothers, report lower quality of life (Caqueo-Urízar et al., 2009; Lima-Rodríguez et al., 2022). The relationship between burden and quality of life is inversely proportional because greater burden is associated with lower perceived well-being (Tristiana et al., 2019; Wang et al., 2025). However, psychoeducation and greater knowledge about Schizophrenia may serve as protective factors that facilitate adaptation and reduce the negative impact of caregiving (Khan and Ghourmulla Alghamdi, 2020).

Despite advances in research on caregivers, studies involving Latin American ethnic populations remain scarce. In Latin America, caregiver burden tends to be higher due to limited institutional support and barriers to accessing mental health services (Caqueo-Urízar et al., 2021). Among the Aymara people, this situation is further complicated by unfavorable socioeconomic conditions, geographical distance from specialized services, and the presence of a unique worldview related to health and illness.

Within Aymara cosmology, mental health is understood through a relational worldview in which individual well-being is closely linked to harmony with the community, nature, and spiritual forces (Caqueo-Urízar et al., 2012b; Valdivia, 2006). Psychological suffering, including severe mental disorders such as Schizophrenia, is often interpreted as a disruption of vital balance or a disconnection from core principles of reciprocity (Ayni) and complementarity (Chachawarmi), which structure social life (Mamani, 2010). From this perspective, illness affects not only the individual but also the communal fabric, and healing processes aim to restore collective harmony rather than treat isolated symptoms.

This cultural interpretation of distress has ambivalent effects. On one hand, community cohesion and cultural beliefs may serve as sources of social support and resilience (Caqueo-Urízar et al., 2018). On the other hand, a preference for traditional healing practices and mistrust of biomedical services may delay diagnosis and timely treatment (Ponce-Correa and Caqueo-Urízar, 2022). Aymara patients also face a double stigma arising from both psychiatric diagnosis and ethnic discrimination, which worsens barriers to treatment access and recovery (Caqueo-Urízar et al., 2022).

In Aymara worldview, personal well-being is grounded in the balance of social relationships, spiritual connections, and ties to nature (Caqueo-Urízar et al., 2012a). Care is associated with values of solidarity, reciprocity, and mutual support within the community, and health is understood holistically by encompassing physical, mental, spiritual, economic, and social dimensions (Valdivia, 2006). However, belonging to an ethnic minority such as the Aymara community may heighten vulnerability for both patient and caregiver, who often experience higher levels of burden and fewer available resources (Caqueo-Urízar et al., 2012a). Supernatural causal beliefs about Schizophrenia also influence treatment adherence and interactions with health systems, which can intensify caregiver distress (Caqueo-Urízar et al., 2015).

Processes of acculturation and migration experienced by Aymara populations generate additional identity-related tensions. Adaptation to the dominant culture often involves the loss of traditional practices and the adoption of more Westernized values (Zapata Silva, 2007). These processes can lead to isolation, discrimination, and reduced community support networks, which negatively affect psychological well-being and increase emotional burden among caregivers.

In response to these challenges, Chile has implemented several intercultural health policies designed to ensure more equitable and culturally appropriate care. Law 20.584 mandates that public health providers offer culturally pertinent services, which include actions such as signage in Indigenous languages and the availability of cultural facilitators (Biblioteca del Congreso Nacional de Chile, 2012). Similarly, the Special Program for Health and Indigenous Peoples (PESPI) aims to reduce health disparities between Indigenous and non-Indigenous populations by promoting community participation and recognizing traditional medicine (Carreño-Calderón, 2021).

Within this context, understanding caregiver burden and quality of life among Aymara caregivers requires an intercultural perspective that considers the sociocultural determinants of caregiving, the central role of the family, and structural barriers to health care access. Based on the evidence presented above, the objective of this study is to analyze caregiver burden and quality of life in individuals caring for persons with schizophrenia, while considering their ethnic background. The study aims to contribute evidence on the ways in which sociocultural and identity-related factors shape caregiving experiences in intercultural contexts.

## Method

### Design

This study employed a non-experimental, cross-sectional, and descriptive-comparative design.

### Sample

The sample consisted of 175 caregivers of individuals diagnosed with Schizophrenia according to DSM-5 criteria. All participants were users of public mental health centers in Arica, Chile. A convenience probabilistic sampling strategy was used for recruitment. Inclusion criteria required participants to be the primary caregiver of a person with schizophrenia for at least 6 months, be 18 years or older, have the capacity and availability to complete the assessment, and provide written informed consent.

### Instruments

Zarit Burden Interview (Zarit et al., 1980): This instrument measures the intensity of caregiver burden in clinical and research settings. It is composed of 22 Likert-type items with five response options (1 = Never, 2 = Rarely, 3 = Sometimes, 4 = Quite often, 5 = Almost always). Scores range from 22 to 110 and classify the caregiver into three levels: absence of burden (22–46), mild burden (47–55), and severe burden (56–110). The scale was validated in Chile by Breinbauer et al. (2009), demonstrating high internal consistency with a Cronbach’s alpha of 0.87.

Schizophrenia Caregiver Quality of Life Questionnaire (S-CGQoL; Richieri et al., 2010): This self-administered questionnaire is designed for caregivers of individuals with Schizophrenia and includes 25 items that measure seven subdimensions: psychological and physical well-being, psychological burden and daily life, relationships with spouse or partner, relationships with family members, relationships with friends, relationships with the psychiatric care team, and material burden. Items are rated on a five-point Likert scale. Scores range from 0, indicating the lowest possible quality of life, to 100, representing excellent quality of life. The questionnaire was adapted into Spanish and validated for Latin American countries by Caqueo-Urízar et al. (2021), showing satisfactory internal consistency (*α* > 0.70 and *ω* > 0.80).

Sociodemographic questionnaire: Information was collected on caregiver and patient characteristics, including sex (male, female), age (45 or younger, older than 45), marital status (single, married, cohabiting, separated, widowed), educational level (university, technical, secondary, primary, no formal education), occupation (homemaker, student, retired, pensioned, self-employed, employed, unemployed, other), religion (Catholic, Evangelical, Jehovah’s Witness, other, no religion), kinship (mother, father, partner, sibling, grandparent, uncle or aunt, institutional worker), economic income (below or equal to minimum wage), ethnicity (Aymara, non-Aymara), and number of hours dedicated to caregiving (few hours per week, several hours per day, most of the day, all the time).

### Procedures

Data collection was conducted by trained psychologists who administered the instruments in face-to-face structured interviews. Caregivers were fully informed about the objectives of the study, potential risks and benefits, inclusion criteria, confidentiality, anonymity, voluntary participation, and their right to withdraw at any time. Those who agreed to participate signed a written informed consent form.

The study received ethical approval from the Scientific Ethics Committee (CEC-UTA) of the Universidad de Tarapacá (18/2019) and from the National Health Service of Chile.

### Statistical analysis

Data processing and statistical analyses were performed using IBM SPSS Statistics version 27 (IBM Corp, 2020). First, descriptive analyses were conducted to characterize the total sample of caregivers and compare Aymara and non-Aymara groups. Frequencies and percentages were calculated for categorical variables, and means and standard deviations were computed for continuous variables.

Pearson’s chi-square tests were used to examine the independence between sociodemographic variables and ethnic group (Aymara or non-Aymara). Independent-samples t tests were applied to assess differences between ethnic groups in caregiver burden scores (Zarit) and quality-of-life scores (S-CGQoL).

Subsequently, two-way univariate analyses of variance (ANOVA) were performed to evaluate the effects of ethnicity and various sociodemographic characteristics of caregivers and patients (age, sex, educational level, kinship, occupation, economic income, religion, and patient age) on caregiver burden, overall quality of life, and their respective subdimensions. Each model included tests of main effects and interactions.

Before conducting the analyses, assumptions of normality and homogeneity of variance were assessed using Q–Q plots and Levene’s test. These assumptions were met in all models. Statistical significance was set at *p* < 0.05.

## Results

Table 1 presents the sociodemographic characteristics of the study participants. The sample consisted of 175 family caregivers of individuals diagnosed with Schizophrenia. The mean age of the caregivers was 57.8 years (SD = 16.3). Most caregivers were women (*n* = 123, 70.7%), and slightly more than half reported being in a partnered relationship (*n* = 86, 50.3%). Regarding educational attainment, 134 participants (78.4%) had completed secondary education, whereas 37 caregivers (21.6%) had not reached this minimum level of schooling.

**Table 1:** Sociodemographic characteristics of the sample.

Variables	Categores	Total sample	Aymara	Non-Aymara	*χ^2^*	*p*
Age	≤45	43 (25.6%)	20 (31.3%)	23 (22.1%)	1.763	0.188
	>45	125 (74.4%)	44 (68.8%)	81 (77.9%)		
Sex	Male	50 (28.7%)	17 (26.2%)	33 (30.3%)	1.956	0.376
	Female	123 (70.7%)	47 (72.3%)	76 (69.7%)		
Partner status	Yes	86 (50.3%)	28 (43.1%)	58 (54.7%)	2.998	0.223
	No	84 (49.1%)	37 (56.9%)	47 (44.3%)		
Completed secondary education	Yes	134 (78.4%)	48 (75.0%)	86 (80.4%)	0.682	0.409
	No	37 (21.6%)	16 (25.0%)	21 (19.6%)		
Occupational status	Not occupied	89 (50.9%)	26 (40.0%)	63 (57.3%)	4.877	0.027
	Occupied	86 (49.1%)	39 (60.0%)	47 (42.7%)		
Religious affiliation	Yes	138 (80.2%)	57 (87.7%)	81 (75.7%)	3.666	0.056
	No	34 (19.8%)	8 (12.3%)	26 (24.3%)		
Caregiver is the patient’s mother	Yes	76 (43.4%)	33 (50.8%)	43 (39.1%)	2.268	0.132
	No	99 (56.6%)	32 (49.2%)	67 (60.9%)		
Income	Equal to or above the minimum wage	48 (29.3%)	20 (31.7%)	28 (27.7%)	0.303	0.582
	Below the minimum wage	116 (70.7%)	43 (68.3%)	73 (72.3%)		
Patient age	≤45	105 (60%)	44 (67.7%)	61 (55.5%)	2.550	0.110
	>45	70 (40%)	21 (32.3%)	49 (44.5%)		
Patient sex	Male	102 (58.3%)	37 (56.9%)	65 (59.1%)	0.079	0.779
	Female	73 (41.7%)	28 (43.1%)	45 (40.9%)		

Minimum wage considered was 357.56 United States dollars (USD). *χ*^2^ = chi-square statistic.

In terms of employment status, 89 caregivers (50.9%) reported having no paid occupation. Most participants reported affiliation with a religion (*n* = 138, 80.2%), and the most common caregiver–patient relationship was mother (*n* = 76, 43.4%). Concerning income, 116 caregivers (70.7%) reported receiving a monthly income below the national minimum wage (357.56 USD). With respect to the characteristics of the care recipients, the mean age of the patients was 40.9 years (SD = 15.6). In total, 102 patients (58.3%) were men and 73 (41.7%) were women.

Chi-square independence tests (Table 1) were conducted to compare the sociodemographic characteristics of caregivers according to ethnic background (Aymara or non-Aymara). The results showed a single statistically significant difference in the variable employment status [*χ*^2^ (1) = 4.877, *p* = 0.027]. A higher proportion of Aymara caregivers held paid employment (60%) compared with non-Aymara caregivers (42.7%). This pattern may reflect cultural differences in work organization and the distribution of family responsibilities, in which Aymara community networks could support greater workforce participation even in the context of long-term caregiving.

Table 2 presents the comparison of mean scores for caregiver burden (Zarit), quality of life (S-CGQoL), and their subdimensions between Aymara and non-Aymara caregivers. The analyses showed statistically significant differences only in two quality-of-life dimensions. In the psychological burden and daily life subdimension, Aymara caregivers obtained higher scores than non-Aymara caregivers, *t*(173) = −1.99, *p* = 0.048, *d* = −0.31, indicating greater emotional strain and a more substantial impact of caregiving on daily activities. In contrast, in the relationship with spouse subdimension, non-Aymara caregivers reported higher mean scores than Aymara caregivers, *t*(168) = 2.04, *p* = 0.043, *d* = 0.32, suggesting a more favorable perception of partner relationships in the context of caregiving.

**Table 2:** Mean differences in caregiver burden (ZARIT) and caregiver quality of life (S_CGCQoL) by ethnic group.

Scales and subscales	Total sample	Aymara	Non-Aymara	*t*	*df*	*p*	*d*
Burden total score (Zarit)	48.2 (18.8)	50.1 (19.4)	47.1 (18.5)	−0.996	164	0.321	−0.160
Quality of life total score (S_CGCQoL)	69.4 (14.0)	70.8 (14.2)	68.6 (13.8)	−0.959	166	0.339	−0.153
Psychological and physical well-being (PsPhW)	14.5 (5.3)	15.1 (5.4)	14.1 (5.3)	−1.184	173	0.238	−0.185
Psychological burden and daily life (PsBDL)	17.8 (7.4)	19.3 (7.9)	17.0 (7.0)	−1.987	173	0.048	−0.311
Relationship with spouse (RS)	7.8 (4.5)	6.9 (4.3)	8.4 (4.5)	2.039	168	0.043	0.323
Relationship with psychiatric team (RPT)	10.7 (4.1)	11.0 (3.9)	10.6 (4.1)	−0.609	173	0.543	−0.095
Relationship with family (RFa)	7.1 (2.8)	7.0 (2.8)	7.1 (2.8)	0.208	173	0.836	0.033
Relationship with friends (RFr)	5.3 (3.0)	5.1 (2.9)	5.4 (3.0)	0.551	172	0.582	0.087
Material burden (MB)	6.5 (3.3)	6.7 (3.5)	6.4 (3.1)	−0.652	171	0.515	−0.102

*d* = effect size (Cohen’s *d*).

The results of the two-way analysis of variance (ANOVA) examining the interaction between ethnicity (Aymara vs. non-Aymara) and various sociodemographic characteristics on caregiver burden, assessed using the Zarit scale, are presented in Table 3. The findings indicate that no statistically significant interaction effects were observed between ethnicity and any of the sociodemographic variables analyzed, including age, sex, presence of a partner, educational level, religion, occupational status, kinship with the patient (mother), income level, patient age, and patient sex (Table 3).

**Table 3:** Interaction effect between ethnicity and sociodemographic characteristics on caregiver burden (ZARIT).

Variable	Sum of squares	*df*	Mean square	*F*	*p*
Age	88.246	1	88.246	0.254	0.615
Sex	333.598	1	333.598	0.951	0.331
Partner status	9.006	1	9.006	0.025	0.874
Completed secondary education	0.165	1	0.165	0.000	0.983
Religious affiliation	225.735	1	225.735	0.649	0.422
Occupational status	76.504	1	76.504	0.214	0.644
Caregiver is the patient’s mother	119.255	1	119.255	0.334	0.564
Income	11.755	1	11.755	0.034	0.855
Patient age	635.836	1	635.836	1.809	0.180
Patient sex	3.937	1	3.937	0.011	0.916

*p* ≤ 0.05 (*); *p* ≤ 0.01 (**).

Subsequently, when analyzing the global scores of caregiver quality of life, a similar pattern emerged. In this case, no statistically significant interaction effects were identified between ethnicity and the sociodemographic characteristics considered. This indicates that caregivers’ perceived quality of life does not vary as a function of these combinations of factors (Table 4).

**Table 4:** Interaction effect between ethnicity and sociodemographic characteristics on caregiver quality of life (S_CGCQoL).

Variable	Sum of squares	*df*	Mean square	*F*	*p*
Age	104.805	1	104.805	0.537	0.465
Sex	350.504	1	350.504	1.810	0.180
Partner status	1.687	1	1.687	0.009	0.925
Completed secondary education	18.905	1	18.905	0.096	0.757
Religious affiliation	0.580	1	0.580	0.003	0.956
Occupational status	333.476	1	333.476	1.710	0.193
Caregiver is the patient’s mother	11.365	1	11.365	0.058	0.810
Income	16.199	1	16.199	0.082	0.774
Patient age	28.941	1	28.941	0.149	0.700
Patient sex	4.815	1	4.815	0.024	0.876

*p* ≤ 0.05 (*); *p* ≤ 0.01 (**).

In addition, the interaction between ethnicity and the sociodemographic variables was assessed for each of the caregiver quality-of-life subdimensions: Psychological and Physical Well-Being (PsPhW), Psychological Burden and Daily Life (PsBDL), Relationship with Spouse (RS), Relationship with Family (RFa), Relationship with Friends (RFr), Relationship with the Psychiatric Team (RPT), and Material Burden (MB). The results indicated a statistically significant interaction between ethnicity and sex in the *Relationship with the Psychiatric Team* subdimension (MS = 108.775, *F* = 6.847, *p* = 0.010), as well as between ethnicity and *Presence of a Partner* in the same subdimension (MS = 124.284, *F* = 8.142, *p* = 0.005). Additionally, religion (MS = 42.816, *F* = 4.033, *p* = 0.046) and patient age (MS = 113.840, *F* = 11.263, *p* < 0.001) showed significant interactions with ethnicity in relation to the *Material Burden* subdimension (Table 5).

**Table 5:** Two-way ANOVA: interaction effects between ethnicity and sociodemographic characteristics on each caregiver quality-of-life subdimension (S-CGCQoL).

Variable	S_CGCQoL means						
	PsPhW	PsBDL	RS	RPT	RFa	RFr	MB
Age	6.290	34.893	2.726	18.356	4.968	0.001	0.159
Sex	0.830	6.646	3.741	108.775**	0.216	3.596	0.330
Partner status	1.219	0.062	30.111	124.284**	0.728	0.395	15.558
Completed secondary education	3.607	14.672	33.829	18.952	0.192	6.935	0.216
Religious affiliation	5.421	4.131	8.090	14.137	12.584	0.939	42.816*
Occupational status	0.160	91.635	27.538	0.025	3.646	0.121	8.269
Caregiver is the patient’s mother	5.398	4.943	29.741	2.732	6.630	0.453	0.250
Income	0.000	3.714	0.309	17.795	0.662	8.193	7.341
Patient age	5.975	9.994	63.281	14.879	0.151	2.034	113.840**
Patient sex	7.862	3.189	1.427	3.343	2.120	0.017	17.616

This table presents the mean squares for each quality-of-life subdimension. *p* ≤ 0.05; *p* ≤ 0.01. The subdimensions are: PsPhW, psychological and physical well-being; PsBDL, psychological burden and daily life; RS, relationship with spouse; RFa, relationship with family; RFr, relationship with friends; RPT, relationship with the psychiatric team; MB, material burden.

## Discussion

This study aimed to analyze the effect of ethnic belonging on the levels of caregiver burden and quality of life among caregivers of individuals with Schizophrenia, providing evidence on how sociocultural and identity-related factors shape the caregiving experience in intercultural contexts. The findings confirm a predominance of female caregivers, particularly mothers over 45 years of age, which aligns with previous evidence indicating that women, and especially mothers, assume the primary caregiving role in families affected by Schizophrenia (Mera et al., 2017; Boyer et al., 2012; Caqueo-Urízar and Gutiérrez-Maldonado, 2006). This gendered burden, more pronounced in the Aymara sample, may be associated with traditional gender roles and with the community-based conception of care characteristic of this ethnic group, where the values of reciprocity, solidarity, and family balance (Valdivia, 2006) reinforce the moral obligation to care for an ill relative, often at the expense of personal well-being.

Additionally, Aymara caregivers exhibited higher levels of labor participation compared to non-Aymara caregivers, despite having significantly lower incomes. This finding relates to the growing rural urban migration of Indigenous populations in search of employment opportunities, a process that has resulted in their incorporation into informal and precarious labor markets (Valenzuela, 2022). This situation reflects a paradox: participation in paid labor can expand financial resources, yet it simultaneously intensifies caregiver burden when paid work coexists with domestic and familial responsibilities, a phenomenon described as double presence.

Despite these socioeconomic disadvantages and the historically documented higher social vulnerability of the Aymara population (Caqueo-Urízar et al., 2012a), the present study found similar levels of burden and quality of life between Aymara and non-Aymara caregivers. This result may reflect structural and cultural transformations relative to earlier research and could plausibly be associated with two complementary phenomena. However, given the cross-sectional design, these interpretations should be understood as contextual hypotheses rather than empirically demonstrated causal explanations.

First, the implementation of intercultural health policies in Chile, such as the Special Program for Health and Indigenous Peoples (PESPI) and the Comprehensive Care Model with an Intercultural Approach from the Ministry of Health (MINSAL, 2018), has contributed to greater access and improved mutual understanding between Indigenous communities and the health system. These policies have promoted more equitable and culturally responsive care, fostering the integration of ancestral knowledge and practices into biomedical services (Carreño-Calderón, 2021). In this sense, the findings are consistent with the possibility that intercultural strategies have contributed to reducing historical gaps and enhancing perceived institutional support among Aymara caregivers. Nevertheless, the present study did not directly assess policy exposure or institutional changes, and therefore this interpretation remains inferential. This articulation between traditional and institutional medicine aligns with international efforts to recognize and strengthen Indigenous healing frameworks, in which health is understood as an integral balance between the body, mind, community, and spirituality.

Second, acculturation and urbanization processes have reshaped traditional cultural patterns, generating a hybridization between Aymara values and the dominant culture (Zapata Silva, 2007). This adaptation may have homogenized caregiving experiences, particularly in urban settings where extended family structures and community networks have been reconfigured. As a result, both Aymara and non-Aymara caregivers face similar challenges in social contexts characterized by weakened community cohesion and increasing dependence on institutional support. Nevertheless, cultural and spiritual practices continue to function as protective mechanisms that strengthen resilience and foster a sense of purpose in the face of mental illness.

Regarding quality of life, significant differences were observed in two subdimensions: Psychological burden and daily life, and Relationship with the spouse. Aymara caregivers reported better psychological well-being, which may be attributed to cultural protective factors such as community resilience, spiritual beliefs, and collective practices of mutual aid that reinforce belonging and social cohesion (van den Hans Berg, 2005). In contrast, the lower scores in spousal relationship among Aymara caregivers suggest that caregiving demands restrict intimacy and shared time with one’s partner, increasing emotional vulnerability (Caqueo-Urízar et al., 2012a). Recent studies highlight that marital satisfaction and strong social support networks are protective factors for caregiver well-being (Stanley and Balakrishnan, 2023).

The significant interactions observed between ethnicity, sex, and presence of a partner across quality-of-life subdimensions indicate that caregiving experiences and perceived support are shaped by relational and cultural factors. Although statistically significant, these interaction effects were generally small to moderate in magnitude, suggesting nuanced rather than structurally large differences between groups. In particular, the caregiver’s sex and the existence of a partner appear to influence how caregiving is organized, how responsibilities are distributed, and how the emotional experience of care unfolds. Women caregivers, who more frequently assume primary caregiving responsibilities, tend to develop greater familiarity with the demands associated with the disorder and with the formal and informal resources available (Khan and Ghourmulla Alghamdi, 2020). However, the ability of mental health services to adapt to diverse cultural and familial contexts remains limited, and strengthening culturally grounded interventions may therefore contribute to enhancing caregiver well-being.

In the Material burden subdimension, the interactions observed between ethnicity and the patient’s age, as well as between ethnicity and the caregiver’s religious affiliation, suggest that perceptions of economic strain are shaped not only by objective caregiving demands but also by the symbolic and cultural frameworks through which these demands are interpreted. Material hardship, in this sense, cannot be understood exclusively as a financial variable; rather, it is embedded in broader systems of meaning that structure responsibility, reciprocity, and moral obligation. Within Aymara cultural traditions, prior ethnographic and qualitative scholarship has conceptualized the relationship with the spiritual realm (Uqu’ pacha) and the principle of reciprocity as moral and cosmological frameworks that organize social life and caregiving practices (Caqueo-Urízar et al., 2012b; Marega and Bah, 2024). From this perspective, caregiving may be integrated into culturally coherent narratives of balance, relational continuity, and communal responsibility, which can shape how material strain is perceived and experienced. These references provide a theoretical lens for interpreting the interaction effects observed in this study. However, it is important to emphasize that such spiritual and cosmological dimensions were not directly assessed in the present research and are introduced here as conceptual frameworks grounded in previous literature rather than as empirically tested mechanisms within this dataset.

This study has methodological limitations that should be considered when interpreting the findings. Although convenience sampling was employed, identifying caregivers of individuals with schizophrenia within Indigenous Aymara communities entails accessing a population that is structurally difficult to reach due to the stigma surrounding severe mental illness, geographic dispersion, and the potential reliance on informal or traditional systems of care. At the same time, recruitment exclusively through public mental health services may have constrained the representativeness of the sample and introduced selection bias, as caregivers outside formal healthcare networks may not be adequately represented. The cross sectional design further limits the ability to draw causal conclusions, and the associations observed between ethnicity, caregiver burden, and quality of life should therefore be understood as context dependent rather than directional. Additionally, the operationalization of ethnicity as a dichotomous variable may not fully reflect the multidimensional and evolving nature of cultural identity. Finally, although only a limited number of interaction effects were statistically significant, the number of analyses conducted raises the possibility of Type I error inflation, and the generally small to moderate effect sizes suggest that these findings should be interpreted cautiously and confirmed through longitudinal and methodologically diversified research.

Overall, the findings are consistent with the possibility of a gradual transformation in caregiving experiences among Aymara caregivers, in which intercultural policies, acculturation processes, and cultural resilience may contribute to explaining the reduced differences observed between ethnic groups. While such contextual dynamics could be associated with a narrowing of disparities in burden and quality of life, they also raise important questions regarding the preservation of traditional community-based support structures that have historically protected Aymara family well-being. Continued progress in mental healthcare will depend on the capacity of health systems to integrate culturally informed approaches to caregiving, advancing toward a broader and contextually grounded understanding of mental health.

## Conclusion

Contrary to earlier studies suggesting that ethnic belonging significantly increased the risk of greater caregiver burden and lower quality of life compared to non-Indigenous caregivers, the present findings indicate that ethnicity exerts a minimal influence, with Aymara and non-Aymara caregivers demonstrating similar experiences. The absence of disparity aligns with the current social landscape, in which greater social integration, improved access to mental health services in primary care, and the development of familial, community, and cultural protective factors have contributed to the homogenization of both populations, reducing structural differences documented more than a decade ago.

The main challenge for Mental Health Services is to continue strengthening the integration of Aymara communities by acknowledging their identity and worldview in addressing their needs. Likewise, programs should be implemented to reinforce protective factors and prevent caregiver burden through continuous counseling, home visits, and support groups.

## Data Availability

The raw data supporting the conclusions of this article will be made available by the authors, without undue reservation.
